# The Treatment of Thoracic Aneurysm

**Published:** 1900-12-08

**Authors:** 


### THE TREATMENT OF THORACIC ANEURYSM.

In the discussion which took place at the last meeting of the Medical Society upon the Diagnosis and Treatment of Thoracic Aneurysm—a discussion which was introduced by Dr. F. de Havilland Hull, and was ultimately adjourned until next week—an interesting communication was made by Dr. Robert Maguire in regard to Lancereaux's method of treatment by the intra-muscular injection of gelatin solution. He said he had treated four cases in this way, all with good results. There could be no question that consolidation had been brought about in these cases. His method was to inject 100 cubic centimetres of absolutely sterile gelatin, of a strength of 2 per cent., made up with a 7 per 1,000 chloride of sodium solution. This was to be injected into the intra-muscular tissue, and he preferred the buttock, taking care to avoid the sciatic nerve. The skin might be frozen with methyl chloride, and the needle should be introduced at least two inches into the muscle. Of course everything must be absolutely sterile. His practice had been to give four injections on four successive days, then to wait for two weeks and repeat the injections, and then to wait again for a further period. There were, he said, certain risks in the method, but they were best minimised by thorough asepsis.

Sir William Broadbent said he had no experience either with gelatin or calcium chloride, and in regard to Tufnell's treatment he thought that iodide of potassium answered every purpose, and rendered Tufnell's method, with its extreme curtailment of fluid, unnecessary. He also pointed out the interesting fact that the great opportunity for successful treatment in cases of aneurysm came, not in the early stage, but later, when the orifice of the aneurysm had become narrowed in proportion to the size of the tumour. Treatment then, which had previously failed, might later be successful.

